# Multi-Parameter Evaluation of Steel Fibre-Reinforced Cementitious Materials for Extrusion-Based 3D Concrete Printing

**DOI:** 10.3390/ma19132921

**Published:** 2026-07-07

**Authors:** Wen Si, Mehran Khan, Ciaran McNally

**Affiliations:** 1 Centre for Critical Infrastructure, School of Civil Engineering, University College Dublin, D04 V1W8 Belfield, Ireland; 2 Construct Innovate, School of Civil Engineering, University College Dublin, D04 V1W8 Belfield, Ireland

**Keywords:** steel fibre, rheology, 3DCP, thixotropy, strength

## Abstract

Extrusion-based three-dimensional concrete printing (3DCP) has emerged as a promising digital construction technology that requires precise control of material rheology and structural performance. This study investigates the influence of steel fibre dosage on the rheological behaviour, mechanical performance, and potential printability of cement-based materials. Mortar mixtures incorporating straight copper-coated steel fibres at dosages from 0 to 1.0% were evaluated. Rheological properties, including yield stress, plastic viscosity, thixotropy, structuration rate, re-flocculation rate, viscosity recovery, and flow index were characterised using rotational rheometry, while compressive and flexural strength were measured at 14 and 28 days. Results show that steel fibres significantly enhance structural build-up and recovery, with both static and dynamic yield stress increasing markedly with fibre dosage. Plastic viscosity and flow behaviour indicate increased resistance to flow at higher contents. Compressive strength exhibits a non-monotonic trend, reaching a maximum increase of approximately 40% at 0.2%, whereas flexural strength is improved at higher fibre dosages due to crack-bridging effects. A normalisation-based composite index was applied to integrate performance indicators. Based on rheology-derived printability implications, a practical working window was proposed, including static yield stress of approximately 280 to 400 Pa, dynamic yield stress of 70 to 160 Pa, plastic viscosity of 7 to 15 Pa·s, re-flocculation rate of 40 to 55 Pa/min and flow index of 0.6 to 1.0. Although higher fibre dosages produced higher composite index values due to dominant rheological enhancement, excessive rheological resistance may reduce practical processability. Based on the proposed working window, 0.2% steel fibre provides the most balanced performance for potential large-scale 3DCP applications.

## 1. Introduction

Extrusion-based three-dimensional concrete printing (3DCP) has rapidly developed as an advanced digital construction technology that enables layer-by-layer fabrication of cementitious materials without the need for conventional formwork, thereby offering significant advantages in terms of geometric freedom, automation and material efficiency [1,2,3]. This technology has demonstrated clear potential to reduce labour demand, construction time, and material waste while enabling the production of complex and customised structural components that are difficult to achieve using traditional casting methods [4]. From a material perspective, 3DCP requires a delicate balance of rheological properties to ensure pumpability, extrudability, and buildability, making rheology a governing factor in successful printing processes [5]. However, despite these advantages, several inherent limitations remain. The absence of conventional reinforcement introduces challenges in achieving sufficient tensile strength and ductility, while weak interlayer bonding and extrusion-induced anisotropy often lead to reduced structural integrity and directional mechanical performance [4,6]. In addition, the demanding and often conflicting rheological requirements can restrict material design flexibility and increase sensitivity to processing conditions, thereby limiting robustness in practical applications [7]. These challenges highlight the need for material modification strategies that can simultaneously enhance fresh-state printability and hardened-state performance, among which the incorporation of fibres has emerged as a promising approach to address these limitations.

Fibres have been widely incorporated into cement-based materials for 3D concrete printing to compensate for the intrinsic brittleness and lack of tensile capacity, and they can be broadly classified into rigid fibres (e.g., steel and glass), synthetic fibres (e.g., polypropylene and polyethylene), and nano-scale fibres or particles (e.g., nano-clay and nano-silica). Rigid fibres, particularly steel fibres, provide high stiffness and strength, enabling effective crack bridging, improved load transfer, and enhanced flexural and tensile performance [8,9,10]. However, they significantly increase yield stress and viscosity, thereby reducing workability and imposing challenges for pumping and extrusion [7,11]. Synthetic fibres offer improve ductility, strain capacity, and crack control with relatively lower impact on flowability, but their lower elastic modulus limits their contribution to structural strength and stiffness [12,13,14,15,16]. Nano-scale fibres and additives primarily act at the microstructural level, refining particle packing and accelerating structuration, which enhances thixotropy and early-age buildability, although their direct contribution to post-cracking mechanical performance remains limited [17,18,19]. Among these fibre types, steel fibres are the most effective in transforming the mechanical behaviour of printed materials from brittle to ductile by promoting fibre–matrix interaction and crack-bridging mechanisms, which are strongly governed by interfacial bond properties and fibre distribution [9,20,21]. Consequently, while all fibre types contribute to improving different aspects of 3DCP performance, steel fibres offer the most direct pathway to overcoming structural limitations, although this comes at the cost of increased rheological complexity that must be carefully controlled.

A growing number of studies have investigated steel fibre-reinforced systems for extrusion-based 3D concrete printing (3DCP), with research primarily focusing on improving structural performance and overcoming the limited reinforcement options available in layer-wise construction. As summarised in Table 1, existing studies have explored a wide range of printable cementitious systems, including ultra-high-performance fibre-reinforced concrete (UHPFRC), high-strength concrete, LC3-based materials and fibre-reinforced mortars, with emphasis on printability, mechanical performance and fibre orientation effects. For instance, a 3D-printable UHPFRC was developed by Arunothayan et al. [22], achieving compressive strengths exceeding 150 MPa and deflection-hardening behaviour suitable for slender structural elements. In a subsequent study, Arunothayan et al. reported that extrusion-induced fibre alignment significantly improved flexural performance and modulus of rupture compared with mould-cast specimens, highlighting the importance of orientation-induced anisotropy [23]. Similarly, Li et al. investigated a two-scale 3D printing strategy for steel fibre-reinforced mortar and demonstrated that controlled fibre arrangement could enhance flexural strength and toughness by more than 100% relative to random fibre distribution [24]. Huang et al. explored magnetically assisted 3D-printed steel fibre-reinforced concrete and showed that active fibre alignment improved crack-bridging efficiency and increased mechanical performance by more than 50% [25]. In addition, bio-inspired Bouligand architectures investigated by Li et al. indicate that tailored fibre orientation and layered configurations can significantly enhance energy absorption capacity and transform failure modes from brittle to ductile [4]. Although these studies consistently demonstrate the effectiveness of steel fibres in enhancing strength, toughness, ductility and anisotropic resistance, the majority focus on hardened-state performance, while comparatively less attention has been devoted to the underlying rheological mechanisms governing fibre dispersion, extrusion behaviour, and process stability.

Recent studies have increasingly recognised the importance of fresh-state properties for successful 3D printing. Arunothayan et al. [26] investigated the rheological behaviour of printable UHPFRC and reported that increasing steel fibre content significantly increased static yield stress, dynamic yield stress and apparent viscosity, thereby improving buildability. Jia et al. [27] demonstrated that steel fibre shape and content strongly influence printability, with excessive fibre contents leading to substantial increases in yield stress and reduced extrudability. Altheoy et al. [28] further showed that the incorporation of double-hooked steel fibres enhanced viscosity, yield stress, pumpability, and buildability of printable UHPFRC. Similarly, Akgümüş et al. [29] reported that waste steel fibres altered rheological resistance and thixotropic behaviour depending on fibre length and dosage. Despite these advances, existing research has primarily focused on individual rheological parameters, such as flowability, yield stress, viscosity or buildability. Comprehensive investigations linking static and dynamic yield stress, viscosity evolution, structural rebuilding behaviour and thixotropic recovery to subsequent mechanical performance remain limited. Furthermore, few studies have proposed a quantitative framework capable of integrating multiple rheological and mechanical indicators to identify optimal steel fibre dosages for 3D printing applications.

Therefore, this study aims to systematically investigate the influence of steel fibre dosage on the rheological and mechanical behaviour of cement-based materials for extrusion-based 3D concrete printing. Particular emphasis is placed on static yield stress, dynamic yield stress, plastic viscosity, structuration rate, re-flocculation rate, viscosity recovery and flow index, together with compressive and flexural strength development. Unlike previous studies that focused primarily on either rheological properties or mechanical performance, this work establishes a comprehensive relationship between fresh-state behaviour and hardened-state performance. In addition, a normalisation-based composite performance framework is proposed to integrate multiple rheological and mechanical indicators and identify an optimal steel fibre dosage for practical printing applications. The findings provide a rheology-driven approach for mixture optimisation and contribute to the development of steel fibre-reinforced materials with balanced printability and structural performance for large-scale 3D concrete printing.

**Table 1:** Summary of steel fibre-reinforced 3DCP studies and positioning of the present work.

Study	Material System	Steel Fibre Type (Dosage)	Fresh Properties Investigated	Printability Assessment	Mechanical Properties	Optimisation/Integrated Evaluation	Main Limitation
Yang et al. [30]	UHPFRC	Straight steel fibre (1.0%)	Limited	√	√	×	Primarily focused on mechanical anisotropy rather than rheological optimisation.
Arunothayan et al. [26]	UHPFRC	Straight steel fibre (0%, 1.0%, 2.0%)	Workability, static yield stress, dynamic yield stress, apparent viscosity	√	Limited	×	Investigated yield stress and viscosity but did not evaluate thixotropic rebuilding or strength development comprehensively.
Jia et al. [27]	High-strength concrete	Straight and hooked-end steel fibre (0%, 0.5%, 1.0%, 1.5%)	Yield stress	√	√	×	Focused on printability and strength; limited rheological characterisation.
Xia et al. [31]	3DCP	Straight steel fibre (0–0.7%)	Flowability	√	√	×	Rheology and mechanical properties evaluated separately; no integrated optimisation methodology.
Altheoy et al. [28]	UHPFRC	Double-hooked steel fibre (1.5%, 3.0%)	Flowability, viscosity, static yield stress, dynamic yield stress, structuration rate (*A*_thix_)	√	√	×	No comprehensive assessment of structural rebuilding behaviour or integrated rheology–mechanical optimisation framework.
Akgümüş et al. [29]	BFS-based 3DPC	Waste steel fibre (0.5%, 1.0%)	Dynamic yield stress, viscosity, structuration rate (*A*_thix_)	√	×	×	Focused on rheology and sustainability; limited mechanical performance evaluation.
Present study	Cement-based mortar	Straight steel fibre (0–1.0%)	Static yield stress, dynamic yield stress, plastic viscosity, structuration rate (*A*_thix_), re-flocculation rate (*R*_thix_), viscosity recovery, flow index (*n*)	Rheology-based printability evaluation	Compressive and flexural strength at 14 d and 28 d	Composite performance index and optimisation framework	Laboratory-scale evaluation; printing validation not included.

## 2. Experimental Methods

### 2.1. Materials

Ordinary Portland cement (CEM II/A-L 42.5 N) conforming to EN 197-1 [32] was used as the primary binder in all mixtures. Limestone filler supplied by Harcourt Technologies Ltd. (HTL, Dublin, Ireland) with a Blaine fineness of 370 ± 20 m^2^/kg and a relative density of 2.7 was incorporated as a supplementary fine material to improve particle packing density and regulate the rheological response of the cementitious system. The chemical compositions of the cement and limestone filler are presented in Table 2. Natural fine aggregate was employed with a maximum particle size of 2 mm and a specific gravity of 2.65, complying with I.S. EN 196-1 [33]. Three chemical admixtures supplied by HTL and conforming to EN 934-2 [34] were incorporated into the mixtures: M5100 as a high-range water-reducing admixture (HRWR), M375 as a water-reducing admixture (WRA), and SDC as a viscosity-modifying admixture (VMA) to enhance mixture stability and shape retention. Reinforcement was provided by straight copper-coated steel fibres with a length of 12 mm and a diameter of 0.39 mm. The fibres were selected to evaluate the influence of steel fibre incorporation on the rheological behaviour and mechanical performance of cement-based materials. All constituent materials and the baseline mixture composition, excluding fibre dosage variations, were supplied by Harcourt Technologies Ltd.

A reference mixture without fibres (Mix No. 1) was first established. Subsequently, mixtures No. 2 to No. 11 were produced by progressively increasing the fibre content from 0.1% to 1.0% by volume of the cementitious matrix (Table 3). While the base mix design was provided by an industrial partner and remains confidential, it was kept constant for all mixtures. Steel fibres were added at the specified dosages without altering the quantities of the other constituents, including cement, limestone filler, water, chemical admixtures, and fine aggregate. Consequently, the total mixture volume increased slightly with increasing fibre dosage, ensuring that any observed changes in fresh or hardened properties could be attributed solely to steel fibres incorporation. 

### 2.2. Research Design and Experimental Workflow

**Figure 1:**
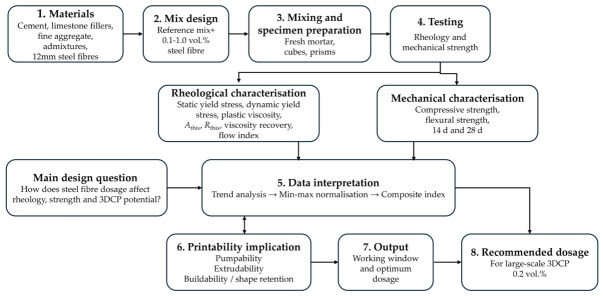
Experimental workflow of this study.

The overall research design and experimental workflow are presented in Figure 1. Steel fibre dosage was selected as the main experimental variable, while the base mixture was kept constant to isolate its influence on fresh and hardened performance. The experimental programme consisted of material preparation, mix design, mixing and specimen preparation, rheological and mechanical characterisation, data interpretation, and printability assessment. The measured rheological and mechanical parameters were further analysed through trend comparison, min–max normalisation, and composite index evaluation. Finally, the results were interpreted in terms of pumpability, extrudability, buildability, and shape retention to establish a practical working window and recommend a suitable steel fibre dosage for large-scale 3D concrete printing.

### 2.3. Test Method

Dry constituents, including cement, limestone filler and fine aggregate, were initially placed in a 10 L Hobart HSM 10 planetary mixer (Hobart UK, Peterborough, UK) and blended at a low rotational speed of 155 ± 5 rpm for 60 s to achieve a uniform dry mixture. Subsequently, the mixing water pre-combined with the chemical admixtures was gradually added over a period of 30 s using a funnel to ensure controlled incorporation and to minimise the risk of segregation. The mixture was then further mixed at the same speed for 60 s to allow complete wetting of all solid particles. Thereafter, the mixing speed was increased to 255 ± 10 rpm and maintained for an additional 60 s to produce a homogeneous mortar. Straight copper-coated steel fibres were then slowly introduced during the final mixing stage to avoid fibre agglomeration, followed by continued mixing for 120 s to ensure uniform fibre dispersion throughout the matrix.

Immediately after mixing, the fresh mixtures were cast into 50 × 50 × 50 mm cube moulds and 40 × 40 × 160 mm prism moulds, which had been pre-coated with a release agent. This specimen size is commonly adopted for printable mortars and cement-based composites and was selected to provide sufficient specimen volume while maintaining practical specimen preparation and curing requirements. Due to the self-compacting nature of the mixtures, no external vibration was applied during casting. The moulds were sealed with plastic sheets to minimise moisture loss during the early stages of hydration. After 24 h, the specimens were demoulded and transferred to a curing tank maintained at 20 ± 2 °C until the designated testing ages of 14 and 28 days. The 14-day compressive strength was considered an early-age strength indicator, as previous studies have used strength development up to 14 days to characterise early-age compressive behaviour in cementitious materials [35,36]. The 28-day strength was used to assess the later-age mechanical performance and overall strength development.

#### 2.3.1. Rheological Test

The rheological properties of the mortar mixtures were evaluated using an Anton Paar MCR 102e SmartPave rotational rheometer (Anton Paar GmbH, Graz, Austria) equipped with a coaxial cylinder Building Material Cell (BMC). The BMC, featuring ribbed internal surfaces to minimise wall slip, had an internal diameter of 70 mm and a height of 100 mm. Immediately after mixing, the fresh mortar was gently transferred into the measuring cell, ensuring a headspace of approximately 50 mm to accommodate material displacement during shearing. A vane-type stirrer with a diameter of 22 mm and a height of 16 mm was employed to apply shear, and was positioned approximately 5 mm above the bottom of the cell to avoid interference with the solid particles, given the maximum aggregate size of 2 mm. All rheological measurements were conducted at a controlled temperature of 20 °C using the rheometer’s temperature-regulation system. Prior to testing, each sample was allowed to rest for 60 s to stabilise and eliminate any transient thermal or structural disturbances.

**Figure 2:**
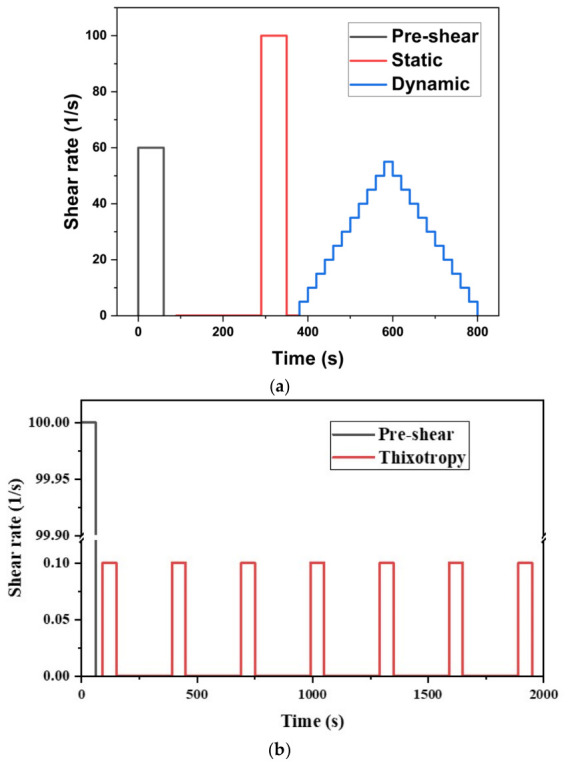
Rheological test protocol: (**a**) static and dynamic test; (**b**) thixotropy test [17,37,38].

Before the main rheological investigation, preliminary repeatability tests were performed to validate the testing protocol and assess measurement consistency. The results demonstrated good repeatability of the rheological parameters. Consequently, single measurements were adopted in the main experimental programme and were considered representative of the rheological response of each mixture. To characterise the time-dependent rheological response relevant to the extrusion-based process, a multi-stage testing protocol was adopted as presented in Figure 2a, representing material conditions during mixing, resting and extrusion [17,37]. The procedure began with a high shear pre-conditioning stage to ensure uniform dispersion of the constituents, followed by a sequence of controlled shear and rest intervals. Thixotropic behaviour was assessed through a cyclic rest-shear approach [38], where the material was first subjected to a pre-shear at 100 s^−1^ for 60 s, and then exposed to repeated low-shear conditions at 0.1 s^−1^ separated by 240 s resting periods (Figure 2b). This protocol enabled the evaluation of structural breakdown under shear and the subsequent rebuilding during rest, providing quantitative insight into the structuration kinetics and time-dependent evolution of the mortar mixtures.

#### 2.3.2. Mechanical Test

Compressive strength was measured in accordance with I.S. EN 196-1 [33] using cube specimens of 50 × 50 × 50 mm. All specimens were cast and cured under identical conditions to ensure consistency. Testing was carried out using a 5980 Series Universal Testing System (Instron, High Wycombe, UK) under load-controlled conditions, with a constant loading rate of 2400 N/s applied until failure. Compressive strength was calculated by dividing the maximum recorded load by the cross-sectional area of the specimen. For each mixture, three cubes were tested and the average value was reported to represent the compressive strength at the specified curing ages.

The flexural performance of the mortar mixtures was assessed in accordance with I.S. EN 1015-11 [39] using prismatic specimens measuring 160 × 40 × 40 mm. Prior to testing, the support span and loading position were marked on each specimen to ensure proper alignment. A three-point bending setup was employed on the same universal testing machine. The loading head was carefully positioned to avoid any unintended preloading. Tests were performed under displacement control at a rate of 0.01 mm/s to allow stable crack propagation and accurate characterisation of the fracture response. Three specimens were tested for each mixture, and the mean value was reported as the representative flexural strength.

## 3. Results

### 3.1. Rheological Results

The rheological properties of the steel fibre-reinforced mortars are presented in Table 4, illustrating the influence of fibre dosage on static yield stress, dynamic yield stress, plastic viscosity, structuration rate and thixotropy. Dynamic yield stress and plastic viscosity were derived from the modified Bingham model, while the flow index (*n*) was obtained from the Herschel–Bulkley model. The fitting coefficients of the rheological models ranged from 0.73 to 0.99 for all mixtures, indicating acceptable to strong agreement between the fitted models and the experimental data. Therefore, the calculated rheological parameters were considered reliable for comparing the influence of steel fibre dosage.

#### 3.1.1. Static Yield Stress

Static yield stress represents the stress required to initiate flow in a material at rest and is commonly used to characterise the structural build-up of cementitious systems [40,41]. Physically, it reflects the degree of interparticle interaction, flocculation, and network formation within the suspension, and is therefore directly associated with shape retention and buildability in extrusion-based processes [42,43,44]. A higher static yield stress indicates a stronger internal structure capable of resisting deformation under self-weight and subsequent layer deposition [45].

**Figure 3:**
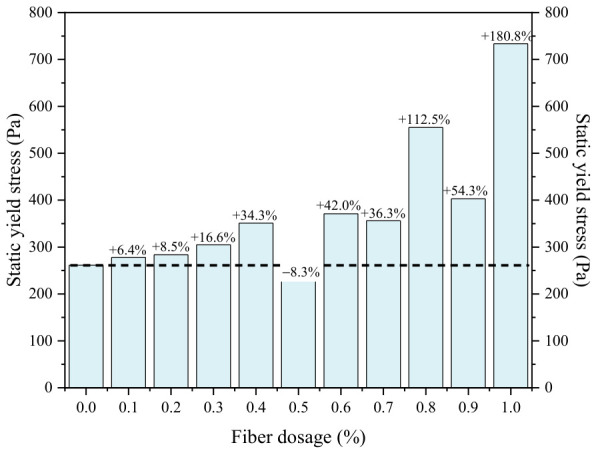
Static yield stress of steel fibre-reinforced mortar as a function of fibre dosage.

The variation in static yield stress with steel fibre dosage is presented in Figure 3. As shown in Figure 3, the reference mixture without fibres exhibited a static yield stress of 261.2 Pa. With the introduction of steel fibres, an overall increasing trend can be observed, although with some fluctuations at intermediate dosages. At low fibre contents (0.1–0.3%), the static yield stress increased gradually from 277.9 Pa to 304.7 Pa, corresponding to an increase of approximately 6–17% compared to the control mixture. This moderate increase suggests that a limited number of fibres contributes to slight enhancement of particle interlocking and structural connectivity without significantly disturbing the flow. A more pronounced increase was observed at 0.4% fibre dosage, where the static yield stress reached 350.9 Pa, representing an increase of approximately 34% relative to the control. This indicates the onset of a more interconnected fibre–particle network. However, a reduction to 239.6 Pa (−8.3%) was recorded at 0.5%, as highlighted in Figure 3. This decrease may be associated with local variations in fibre distribution and fibre–fibre interactions within the cementitious matrix, which can influence the development of the flocculated network and temporarily reduce its resistance to deformation. Beyond this point, the static yield stress increased significantly with further fibre addition. At 0.6% and 0.7%, values of 371.0 Pa and 356.1 Pa were obtained, indicating recovery and stabilisation of the structural network. A substantial increase was observed at 0.8%, where the yield stress reached 555.1 Pa, more than doubling the control value. The maximum value of 733.59 Pa was achieved at 1.0% fibre dosage, corresponding to an increase of approximately 181% compared to the reference mixture. This sharp increase demonstrates that high fibre contents lead to a dense and highly interconnected network that strongly resists deformation.

The observed trend can be attributed to the combined effects of fibre–fibre interaction, fibre–particle interaction, and mechanical interlocking. At low dosages, fibres act as discrete inclusions, slightly enhancing structural build-up. As the dosage increases, fibres begin to form a continuous network that restricts particle mobility and promotes flocculation, thereby increasing the static yield stress. At higher dosages, the dominant mechanism becomes fibre entanglement and contact friction, which significantly increases resistance to flow initiation. However, the non-monotonic behaviour at intermediate dosage indicates that fibre dispersion quality plays a critical role in determining the effectiveness of this reinforcement mechanism.

#### 3.1.2. Dynamic Yield Stress

Dynamic yield stress corresponds to the stress required to sustain flow once the material structure has been broken down under shear and is typically derived from steady-state flow conditions [42,46,47]. It reflects the resistance of the material during continuous deformation and is therefore closely associated with pumpability and extrudability in extrusion-based 3DCP. In contrast to static yield stress, which governs shape retention at rest, dynamic yield stress controls the ease of material transport and deposition through the printing system. An appropriate balance between these two parameters is essential to ensure stable extrusion without blockage while maintaining sufficient structural integrity after deposition.

**Figure 4:**
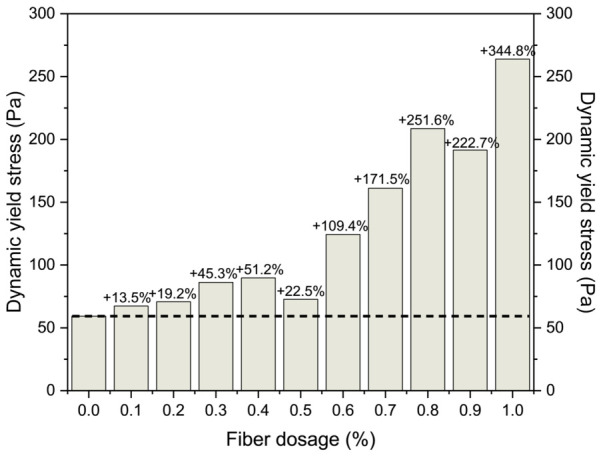
Dynamic yield stress of steel fibre-reinforced mortar as a function of fibre dosage.

The variation in dynamic yield stress with steel fibre dosage is presented in Figure 4. The control mixture exhibited a dynamic yield stress of 59.3 Pa. At low fibre contents (0.1–0.4%), a gradual increase was observed with values rising from 67.3 Pa to 89.8 Pa, corresponding to an increase of approximately 14–51% compared to the reference mixture. This increase indicates that even small amounts of steel fibres introduce additional resistance to flow due to fibre–particle interactions and increased internal friction. At 0.5% fibre dosage, a reduction in dynamic yield stress to 72.7 Pa was recorded, deviating from the increasing trend. This decrease suggests a temporary disruption of the flow network, likely associated with non-uniform fibre dispersion or localised fibre clustering, which can reduce the effective stress transfer during shear. A similar phenomenon has been reported in fibre-reinforced systems where heterogeneity in fibre distribution leads to inconsistent rheological response [48]. Beyond 0.5%, a significant increase in dynamic yield stress was observed. At 0.6% and 0.7%, the values increased to 124.3 Pa and 161.1 Pa, respectively, indicating a transition towards a fibre-dominated flow regime. A more pronounced increase occurred at 0.8%, where the dynamic yield stress reached 208.6 Pa, more than three times the control value. The maximum value of 264.0 Pa was recorded at 1.0%, corresponding to an increase of approximately 345% relative to the reference mixture. This sharp rise reflects the formation of a dense fibre network that significantly restricts flow under shear.

The observed trend demonstrates that the influence of steel fibres on dynamic yield stress evolves from a moderate increase at low dosages to a rapid escalation at higher contents. At low fibre concentrations, fibres act as isolated inclusions that slightly increase viscous resistance. As the fibre dosage increases, fibre–fibre interactions become more frequent, leading to the development of a contact network that prevents flow. Once a critical concentration is reached, this network becomes continuous, resulting in a substantial increase in flow resistance. This behaviour is consistent with theoretical models describing the percolation of rigid fibre networks, where the yield stress increases sharply once fibre contacts dominate the system [45]. From a 3D-printing perspective, the increase in dynamic yield stress enhances extrusion stability by preventing segregation and filament collapse during deposition. However, excessively high values, particularly at fibre dosages above 0.8%, may lead to increased pumping pressure and a higher risk of blockage, indicating the need for optimisation of fibre content to balance extrudability and buildability.

#### 3.1.3. Plastic Viscosity

Plastic viscosity represents the slope of the shear stress–shear rate relationship after yielding and characterises the resistance of a material to flow under continuous shear [45,49]. It reflects the internal friction within the suspension, arising from particle–particle interactions, hydrodynamic forces, and energy dissipation during flow. In cementitious systems, plastic viscosity governs the rate of deformation once flow has been initiated and is therefore closely related to flow stability and material transport. In the extrusion-based 3DCP, plastic viscosity is a key parameter controlling pumpability and filament continuity. A sufficiently high viscosity is required to maintain coherent extrusion and prevent segregation, whereas excessively high viscosity can increase pumping pressure and hinder smooth material delivery [31,50].

**Figure 5:**
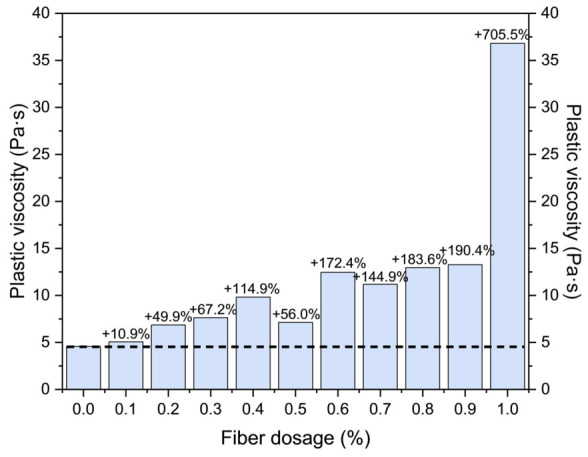
Plastic viscosity of steel fibre-reinforced mortar as a function of fibre dosage.

The variation in plastic viscosity with steel fibre dosage is presented in Figure 5. The reference mixture exhibited a plastic viscosity of 4.6 Pa⋅s. With the introduction of steel fibres, an overall increasing trend is observed, although with noticeable fluctuations at intermediate dosages. At low fibre contents (0.1–0.4%), plastic viscosity increased progressively from 5.1 Pa⋅s to 9.8 Pa⋅s, corresponding to an increase of approximately 11–115% compared to the control mixture. This increase reflects the additional resistance to flow induced by fibre inclusion, which enhances internal friction and energy dissipation during shear. At 0.5% fibre dosage, a reduction to 7.1 Pa·s was observed, deviating from the overall increasing trend. This behaviour is consistent with the corresponding yield stress response and may reflect local variations in fibre distribution and fibre–fibre interactions within the matrix. However, beyond this dosage, plastic viscosity increased again, reaching 12.5 Pa⋅s at 0.6% and remaining relatively high at 0.7% and 0.8% (11.2–13.0 Pa⋅s). A moderate increase was further observed at 0.9% (13.3 Pa⋅s), followed by a sharp rise to 36.8 Pa⋅s at 1.0%, representing an increase in more than 700% relative to the control mixture. This significant increase indicates a transition to a highly resistant flow regime.

The evolution of plastic viscosity differs from that of yield stress in that it is primarily governed by energy dissipation mechanisms during flow rather than the formation of a load-bearing network at rest. At low fibre contents, fibres act as discrete obstacles that increase viscous drag by disturbing the flow field and enhancing particle–fluid interactions. As the fibre dosage increases, fibre–fibre interactions become more frequent, leading to increased collision, sliding, and friction during shear. At higher dosages, fibre entanglement and interaction with the surrounding matrix create a complex flow network that significantly increases resistance to deformation. Unlike yield stress, which is strongly associated with network percolation, plastic viscosity is more sensitive to the dynamic interactions and mobility constraints imposed by fibres during flow.

#### 3.1.4. Thixotropy

The thixotropic behaviour of cementitious materials can be described through two complementary mechanisms, namely the structuration rate (*A*_thix_) and the re-flocculation rate (*R*_thix_), which together govern the time-dependent rebuilding of the internal structure after shear [51,52,53,54]. The structuration rate represents the progressive increase in static yield stress with time and is primarily associated with long-term structural build-up driven by hydration reactions, particle interactions, and the formation of a percolated network. In contrast to the rapid recovery captured by *R*_thix_, *A*_thix_ reflects a slower but sustained stiffening process that contributes to the development of mechanical stability in fresh cementitious systems. In extrusion-based 3DCP, *A*_thix_ is directly related to the ability of the deposited filament to gain sufficient rigidity over time to support subsequent layers, thereby influencing buildability and height retention [55,56].

**Figure 6:**
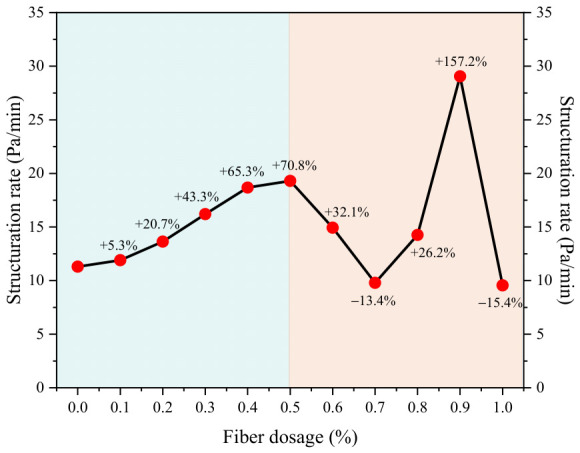
Structuration rate (*A*_thix_) of steel fibre-reinforced mortar as a function of fibre dosage.

The evolution of structuration rate with steel fibre dosage is presented in Figure 6. The reference mixture exhibited a structuration rate of 11.3 Pa/min. At low fibre contents (0.1–0.5%), a consistent increase was observed, with values rising to 19.3 Pa/min at 0.5%, corresponding to an increase of approximately 70.8%. This progressive increase indicates that the incorporation of steel fibres enhances the long-term structural build-up, which can be attributed to increased particle confinement and enhanced interparticle interactions that promote hydration-induced network formation. In this range, fibres act as rigid inclusions that restrict particle mobility and facilitate the accumulation of flocculated structures over time. At 0.4% and 0.5%, the structuration rate reached 18.7 Pa/min and 19.3 Pa/min, respectively, marking a transition where fibre–particle interactions significantly contribute to the structural evolution of the system. However, beyond this dosage, a reduction in structuration rate was observed. At 0.6% and 0.7%, the values decreased to 14.9 Pa/min and 9.8 Pa/min, corresponding to reductions of −32.1% and −13.4% relative to the peak region. This decline suggests that excessive fibre content may hinder the continuous development of the hydration-driven network, likely due to fibre interference and local heterogeneity that limit effective particle rearrangement and bonding. A distinct increase was observed at 0.9%, where the structuration rate reached 29.1 Pa/min, representing an increase of 157.2% compared to the reference mixture. This sharp rise indicates the formation of a highly constrained internal structure, where fibre–fibre and fibre–particle interactions significantly accelerate the accumulation of structural resistance over time. However, at 1.0%, the structuration rate decreased again to 9.6 Pa/min, indicating instability in the structural build-up process at very high fibre contents.

The non-monotonic evolution of structuration rate reflects the competition between hydration-driven structuration and fibre-induced constraints. At low to moderate dosages, steel fibres enhance structural build-up by promoting particle interaction and limiting structural relaxation. As the dosage increases, fibre interference and dispersion limitations may reduce the efficiency of hydration product connectivity, leading to a temporary decrease in structuration rate. At higher dosages, localised fibre networks can accelerate structural stiffening, although this effect becomes unstable when excessive fibre content disrupts the continuity of the matrix.

The re-flocculation rate (*R*_thix_) characterises the rapid rebuilding of the internal microstructure immediately after the cessation of shear and is typically quantified from the recovery of shear stress under low shear conditions [53,55]. It reflects the short-term re-agglomeration of particles driven by interparticle attractive forces, such as van der Waals interactions and electrostatic effects. In contrast to the structuration rate, which is governed by slower hydration-driven processes, *R*_thix_ captures the instantaneous structural recovery that occurs immediately after extrusion. This parameter is therefore critical in determining the early-stage stability of deposited filaments in extrusion-based 3DCP. In practical terms, a higher re-flocculation rate enables the material to quickly regain sufficient rigidity after deposition, reducing filament spreading and enhancing shape retention. As reported in previous studies, *R*_thix_ is typically one order of magnitude higher than the structuration rate, indicating its dominant role in controlling the initial stability of printed layers [57]. However, excessively high *R*_thix_ may reduce the ability of successive layers to form adequate interfacial bonding due to rapid stiffening, highlighting the need for a balanced rheological response.

**Figure 7:**
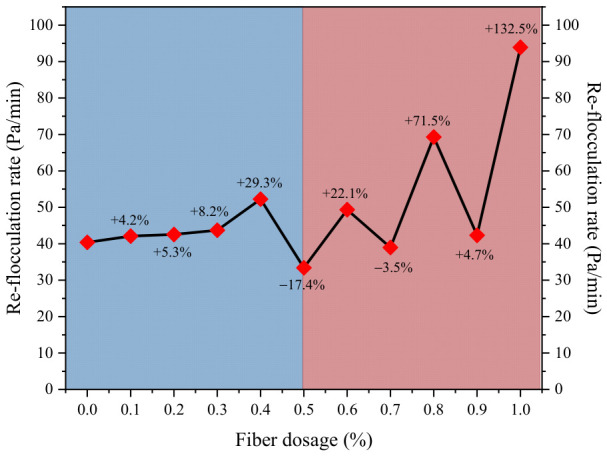
Re-flocculation rate (*R*_thix_) of steel fibre-reinforced mortar as a function of fibre dosage.

The variation in re-flocculation rate with steel fibre dosage is presented in Figure 7. The reference mixture exhibited a re-flocculation rate of 40.4 Pa/min. At low fibre contents (0.1–0.4%), a gradual increase was observed, with values rising to 52.2 Pa/min at 0.4%, corresponding to an increase of approximately 29.3%. This trend indicates that the introduction of steel fibres enhances the rapid recovery of the microstructure, likely due to increased particle confinement and enhanced interparticle attraction facilitated by the presence of rigid inclusions. At 0.5% fibre dosage, a significant reduction to 33.4 Pa/min (−17.4%) was observed, indicating a temporary reduction in the re-flocculation rate. This behaviour may be associated with local variations in fibre distribution and fibre–fibre interactions within the matrix, which can influence the rapid rebuilding of the particle network after shear. A similar behaviour was observed at 0.7% (39.0 Pa/min, −3.5%), indicating that intermediate fibre contents may introduce instability in short-term structural recovery. Beyond this region, the re-flocculation rate increased markedly. At 0.8%, the value reached 69.3 Pa/min (+71.5%), indicating a substantial enhancement in rapid structural rebuilding. The highest value of 93.9 Pa/min was recorded at 1.0%, corresponding to an increase of 132.5% relative to the reference mixture. This significant increase suggests that at high fibre contents, fibre–fibre interactions and contact networks contribute to rapid stress recovery, effectively restricting particle mobility immediately after shear cessation.

The overall trend demonstrates that the re-flocculation behaviour is governed by a combination of particle-level interactions and fibre-induced constraints. At low fibre dosages, fibres promote re-agglomeration by enhancing particle confinement and increasing the frequency of particle contacts. At intermediate dosages, dispersion-related issues may disrupt the continuity of the particle network, reducing the efficiency of structural recovery. At higher dosages, the formation of a dense fibre network accelerates stress recovery through mechanical restriction and frictional interactions, leading to a rapid increase in *R*_thix_. From a 3D-printing perspective, the increase in re-flocculation rate at higher fibre contents contributes to improved filament stability and reduced deformation immediately after deposition. However, the non-monotonic behaviour observed at intermediate dosages highlights the importance of fibre dispersion in achieving consistent rheological performance.

#### 3.1.5. Viscosity Recovery

The thixotropic behaviour of cementitious materials can also be characterised through viscosity recovery, which quantifies the ability of the material to rebuild its internal structure following shear-induced breakdown [58,59]. This parameter is obtained from a step-wise shear protocol, where the material experiences sequential low–high–low shear stages. The corresponding viscosity response reflects three distinct regimes, namely the initial structural state, structural breakdown under high shear, and subsequent structural regeneration. The viscosity recovery is defined as the difference between the minimum viscosity after breakdown and the maximum viscosity after rebuilding and thus represents the extent of structural regeneration over a short time scale. Physically, it reflects the reformation of particle–particle and particle–fibre interactions, as well as the recovery of the suspension network after shear. Viscosity recovery is directly related to the ability of the extruded filament to maintain its geometry after deposition in the 3DCP. A higher recovery indicates a stronger capacity for rapid structural regeneration, which contributes to filament cohesion, reduced spreading, and improved dimensional stability [17,60]. However, excessive recovery may also lead to premature stiffening, which can negatively affect interlayer bonding and extrusion continuity. Therefore, viscosity recovery provides an important complementary measure to yield stress and thixotropy in assessing printability.

**Figure 8:**
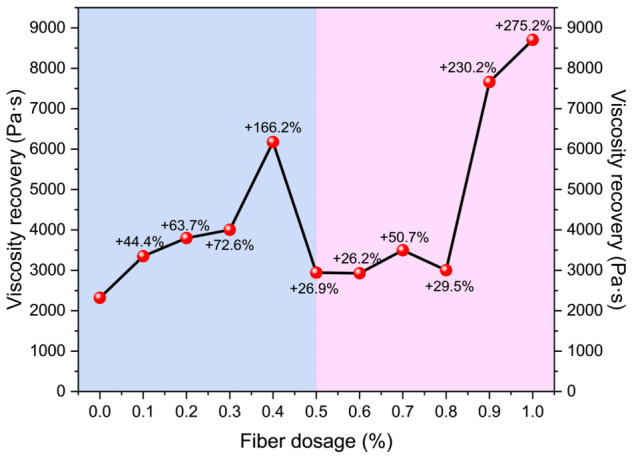
Viscosity recovery of steel fibre-reinforced mortar as a function of fibre dosage.

The evolution of viscosity recovery with steel fibre dosage is presented in Figure 8. The reference mixture exhibited a viscosity recovery of 2320.0 Pa⋅s. At low fibre contents (0.1–0.4%), a consistent increase was observed, reaching 6175.9 Pa⋅s at 0.4%, corresponding to an increase of 166.2%. This progressive increase indicates that the incorporation of steel fibres significantly enhances the ability of the material to regenerate its internal structure after shear. In this range, fibres act as rigid constraints that promote the re-establishment of particle networks and increase resistance to deformation during the recovery stage. At 0.5% and 0.6%, a notable reduction in viscosity recovery was observed, with values of 2943.1 Pa⋅s and 2928.2 Pa⋅s, respectively. Despite remaining above the reference level, this reduction suggests a temporary weakening of the recovery mechanism, likely due to fibre interference or non-uniform dispersion, which can hinder the efficient reformation of the particle network. A partial recovery was observed at 0.7% and 0.8%, with values of 3496.1 Pa⋅s and 3003.3 Pa⋅s, indicating moderate structural rebuilding.

A significant increase occurred at higher fibre contents. At 0.9%, viscosity recovery reached 7660.3 Pa⋅s, corresponding to an increase of 230.2% relative to the control mixture. The maximum value of 8703.7 Pa⋅s was recorded at 1.0%, representing an increase of 275.2%. This sharp increase indicates the formation of a highly constrained network where fibre–fibre interactions dominate the recovery process, leading to rapid and substantial structural regeneration after shear. The observed trend reflects the transition from particle-dominated recovery at low fibre contents to fibre-dominated interactions at higher dosages. Unlike yield stress and plastic viscosity, which primarily describe resistance during flow, viscosity recovery captures the post-shear rebuilding capability, highlighting the dynamic restructuring behaviour of fibre-reinforced systems.

#### 3.1.6. Flow Index

The flow index (*n*), derived from the Herschel–Bulkley model, characterises the shear-dependent flow behaviour of cementitious materials and provides insight into the deviation from ideal Bingham behaviour. A value of *n* < 1 indicates shear-thinning behaviour, where the apparent viscosity decreases with increasing shear rate due to progressive breakdown and rearrangement of particle flocculations. In contrast, *n* > 1 represents shear-thickening behaviour, where hydrodynamic interactions and particle clustering lead to an increase in resistance at higher shear rates. As such, the flow index reflects the balance between structural breakdown and dynamic particle interactions under shear [61,62]. The flow index plays a critical role in governing flow stability and material transport. Shear-thinning behaviour is generally beneficial for pumpability and extrudability, as it facilitates flow under high shear conditions within the pumping and extrusion system. Conversely, moderate shear-thickening behaviour can enhance filament cohesion and stability after extrusion by increasing resistance under deformation [63]. Therefore, the transition between shear-thinning and shear-thickening regimes is particularly relevant for achieving a balanced rheological response suitable for printing.

**Figure 9:**
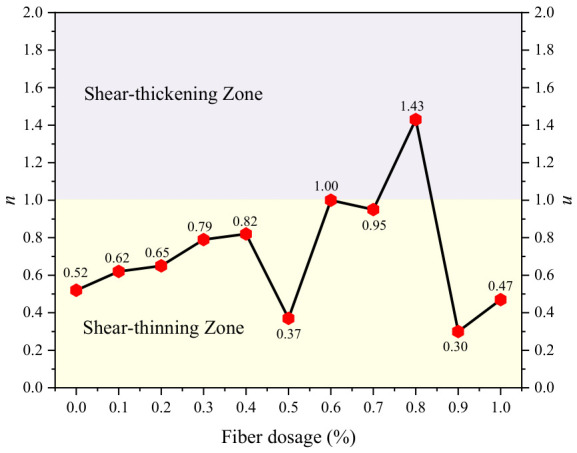
Flow index (*n*) of steel fibre-reinforced mortar as a function of fibre dosage.

The variation in flow index with steel fibre dosage is presented in Figure 9. The reference mixture exhibited a flow index of 0.52, indicating pronounced shear-thinning behaviour. At low fibre contents (0.1–0.4%), the flow index increased progressively from 0.62 to 0.82, while remaining within the shear-thinning regime (*n* < 1). This gradual increase suggests that the addition of steel fibres reduces the degree of shear thinning by introducing additional resistance to flow, likely due to fibre–particle interactions and increased internal friction. At 0.5%, a sharp decrease in the flow index to 0.37 was observed, indicating enhanced shear-thinning behaviour. This reduction is consistent with the behaviour observed in other rheological parameters and may be attributed to localised structural disruption, which facilitates flow under shear. A transition point was observed at 0.6%, where the flow index reached 1.00, indicating a shift towards Bingham-type behaviour. Beyond this dosage, the system exhibited alternating behaviour between shear-thinning and shear-thickening regimes. At 0.8%, a significant increase to 1.43 was recorded, indicating the strongest shear-thickening behaviour.

The observed non-monotonic variation in flow index reflects the complex interplay between particle rearrangement, fibre interaction, and flow-induced structuring. From a mechanistic perspective, the transition towards shear-thickening behaviour at higher fibre contents may be attributed to increased hydrodynamic interactions and contact between fibres, which restrict flow under shear. In contrast, the return to shear-thinning behaviour at certain dosages suggests that fibre dispersion and orientation play a critical role in determining the effective flow regime.

### 3.2. Mechanical Results

The mechanical performance results of steel fibre-reinforced mortars are summarised in Table 5, including compressive and flexural strengths at 14 and 28 days.

#### 3.2.1. Compressive Strength

**Figure 10:**
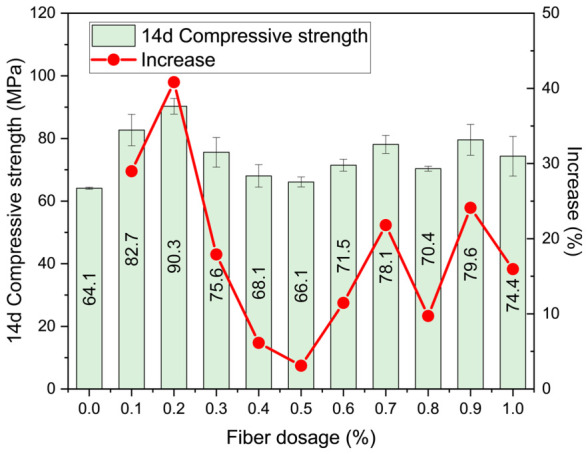
Compressive strength and corresponding increase in steel fibre-reinforced mortar at 14 days.

**Figure 11:**
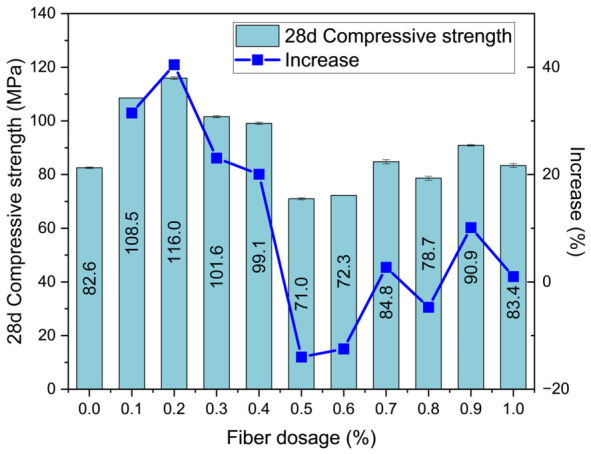
Compressive strength and corresponding increase in steel fibre-reinforced mortar at 28 days.

The compressive strength development of steel fibre-reinforced mortars at 14 days and 28 days are presented in Figure 10 and Figure 11, respectively, together with the corresponding increase ratio relative to the reference mixture. The results indicate a non-monotonic dependence of compressive strength on fibre dosage, reflecting the balance between fibre reinforcement and matrix disruption.

At 14 days (Figure 10), the reference mixture exhibited a compressive strength of 64.1 MPa. With the incorporation of steel fibres, a significant increase was observed at low dosages, reaching 82.7 MPa at 0.1% and a maximum value of 90.3 MPa at 0.2%, corresponding to an increase of 40.8%. This indicates that a moderate fibre dosage is most effective in enhancing early-age strength. Beyond this point, the compressive strength decreased to 75.6 MPa at 0.3% and further to 68.1 MPa and 66.1 MPa at 0.4% and 0.5%, respectively, with the minimum value occurring at 0.5%. At higher fibre contents, the strength partially recovered, reaching 78.1 MPa at 0.7% and 79.6 MPa at 0.9%, before decreasing slightly to 74.4 MPa at 1.0%.

At 28 days (Figure 11), the reference mixture exhibited a compressive strength of 82.6 MPa. A similar trend was observed, with the strength increasing to 108.5 MPa at 0.1% and reaching a maximum of 116.0 MPa at 0.2%, corresponding to the highest increase of 40.5%. However, beyond this dosage, a more pronounced reduction was observed compared to the 14-day results. The strength decreased to 101.6 MPa at 0.3% and 99.1 MPa at 0.4%, followed by a significant drop to 71.0 MPa at 0.5%, which represents the minimum value and corresponds to a reduction of −14.0% relative to the control mixture. Although a partial recovery was observed at 0.6% and 0.7%, the strength remained lower than the reference for several intermediate dosages. At higher fibre contents, the strength increased again to 90.9 MPa at 0.9%, before slightly decreasing to 83.4 MPa at 1.0%.

The observed trends at both curing ages can be attributed to the dual role of steel fibres. At low dosages, fibres enhance compressive strength by improving stress transfer, limiting microcrack propagation, and promoting a more uniform stress distribution. This effect is most pronounced at 0.2%, where fibre dispersion is likely optimal. As the fibre content increases, the negative effects associated with reduced workability, increased porosity and potential fibre clustering become dominant, leading to strength reduction, particularly at 0.5%. At higher dosages, fibre–fibre interaction and confinement effects contribute to partial strength recovery, although the presence of matrix discontinuities limits further improvement.

#### 3.2.2. Flexural Strength

**Figure 12:**
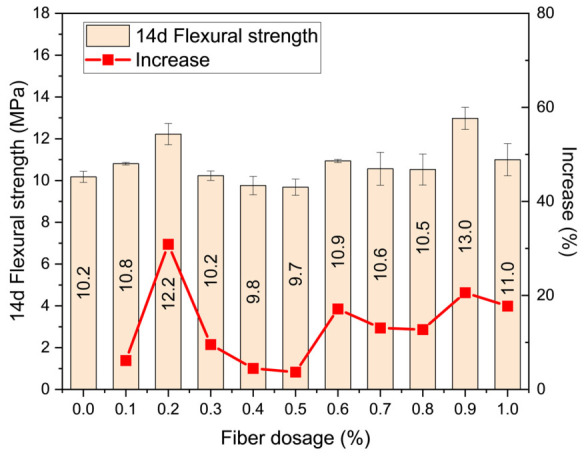
Flexural strength and corresponding increase in steel fibre-reinforced mortar at 14 days.

**Figure 13:**
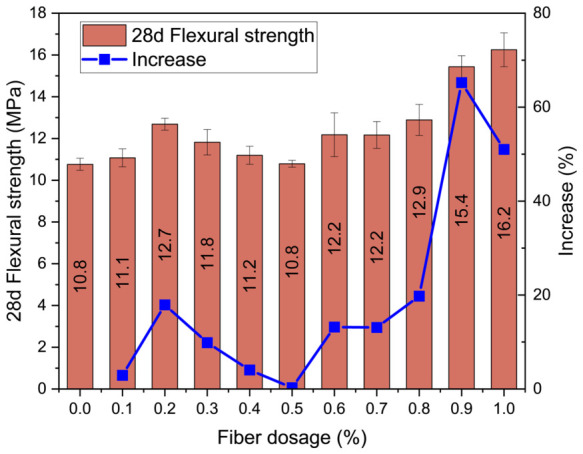
Flexural strength and corresponding increase in steel fibre-reinforced mortar at 28 days.

The flexural strength of steel fibre-reinforced mortars at 14 days and 28 days are presented in Figure 12 and Figure 13, respectively, together with the corresponding increase ratio relative to the reference mixture. Compared to compressive strength, flexural strength exhibits a more consistent enhancement with fibre addition, reflecting the dominant role of fibres in crack bridging and post-cracking resistance.

At 14 days (Figure 12), the reference mixture exhibited a flexural strength of 10.2 MPa. With the incorporation of steel fibres, the strength increased to 10.8 MPa at 0.1% and reached a maximum of 12.2 MPa at 0.2%, corresponding to the highest increase of 30.87%. This indicates that a relatively low fibre dosage is sufficient to significantly improve early-age flexural performance. Beyond this point, the flexural strength decreased to 10.2 MPa at 0.3% and further to 9.8 MPa and 9.7 MPa at 0.4% and 0.5%, respectively, with the minimum value observed at 0.5%. At higher fibre contents, the strength gradually recovered, reaching 10.9 MPa at 0.6% and stabilising in the range of 10.5–11.0 MPa for 0.7–1.0%, with moderate increases up to 21.80%.

At 28 days (Figure 13), the reference mixture exhibited a flexural strength of 10.8 MPa. A similar initial increase was observed, with values of 11.1 MPa at 0.1% and 12.7 MPa at 0.2%, corresponding to an increase of 17.9%. However, the trend diverged at higher fibre dosages. After a slight decrease at intermediate dosages (0.3–0.5%), the flexural strength increased significantly beyond 0.6%, reaching 12.2 MPa at 0.6% and continuing to rise to 12.9 MPa at 0.8%. The most pronounced enhancement was observed at 0.9%, where the flexural strength reached 15.4 MPa, corresponding to the highest increase of 65.2%. At 1.0%, the strength remained high at 16.2 MPa, with an increase of 51.0%, indicating sustained improvement at high fibre contents.

The observed trends highlight the distinct role of steel fibres in flexural behaviour compared to compressive performance. At low dosages, fibres effectively bridge microcracks and delay crack propagation, resulting in a rapid increase in flexural strength. The reduction observed at intermediate dosages (around 0.4–0.5%) may be associated with local variations in fibre distribution and fibre–fibre interactions, which can reduce the efficiency of stress transfer across cracks. At higher dosages, the formation of a more continuous fibre network enhances crack bridging and pull-out resistance, leading to significant improvements in flexural strength, particularly at 28 days. The difference between early-age and later-age behaviour suggests that the contribution of steel fibres becomes more pronounced as the matrix matures and the fibre–matrix bond strength increases. As a result, while optimal performance at 14 days is achieved at a relatively low fibre content (0.2%), higher fibre dosages (0.9–1.0%) are more effective in maximising flexural strength at 28 days.

#### 3.2.3. Curing Age Effect

**Figure 14:**
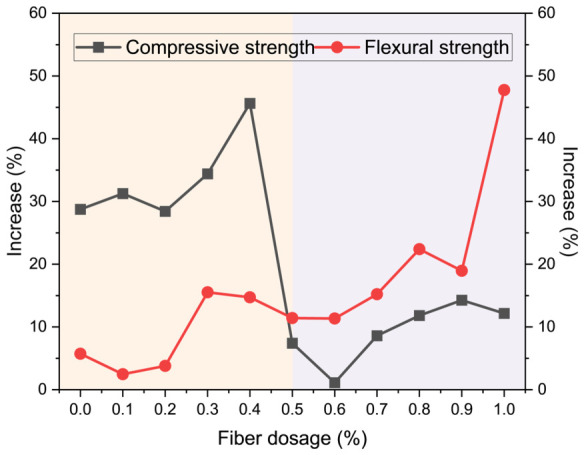
Increase in compressive and flexural strength from 14 to 28 days as a function of steel fibre dosage.

The evolution of mechanical performance from 14 to 28 days is illustrated in Figure 14, where the increase ratio in compressive and flexural strength is presented as a function of steel fibre dosage. The results show distinct trends for compressive and flexural behaviour, reflecting the different governing mechanisms at later ages.

For compressive strength, the increase is generally stable at low fibre contents, with values around 28–31%. A pronounced peak is observed at 0.4%, where the increase reaches the maximum value of 45.6%, indicating an optimal balance between matrix densification and fibre confinement at this dosage. In contrast, a sharp reduction occurs at 0.6%, where the increase drops to a minimum of 1.1%, suggesting that the contribution of continued hydration to strength development is significantly hindered, likely due to matrix discontinuity and fibre interference. At higher fibre contents, the increase partially recovers but remains moderate, indicating limited additional contribution to compressive strength gain.

In contrast, flexural strength exhibits a markedly different evolution. The increase is relatively low at low fibre contents, remaining below 6%, indicating that early-age fibre contribution dominates flexural behaviour. However, a significant enhancement is observed at higher fibre dosages, particularly at 1.0%, where the increase reaches the maximum value of 47.8%. This substantial improvement highlights the increasing effectiveness of fibre bridging and pull-out resistance as the matrix matures and fibre–matrix bonding strengthens.

The contrasting trends between compressive and flexural strength highlight the different roles of steel fibres in mechanical performance. While compressive strength development is primarily governed by matrix densification and is sensitive to fibre-induced defects at higher dosages, flexural strength is strongly influenced by fibre bridging and pull-out resistance, which become more effective as the matrix matures. As a result, the benefit of steel fibres is more pronounced in flexural performance at later ages, particularly at higher fibre contents.

## 4. Discussion

### 4.1. Normalisation Analysis

To enable direct comparison between rheological and mechanical parameters with different units and magnitudes, all measured properties were first normalised using a min–max scaling approach. Each parameter was transformed into a dimensionless value between 0 and 1, where 0 corresponds to the minimum and 1 to the maximum value within the dataset. Subsequently, negative indicators including plastic viscosity, structuration rate and flow index were inverted to ensure that higher values consistently represent better performance. This unified normalisation framework allows all parameters to be evaluated on the same scale and enables a comprehensive assessment of the influence of steel fibre dosage.

**Figure 15:**
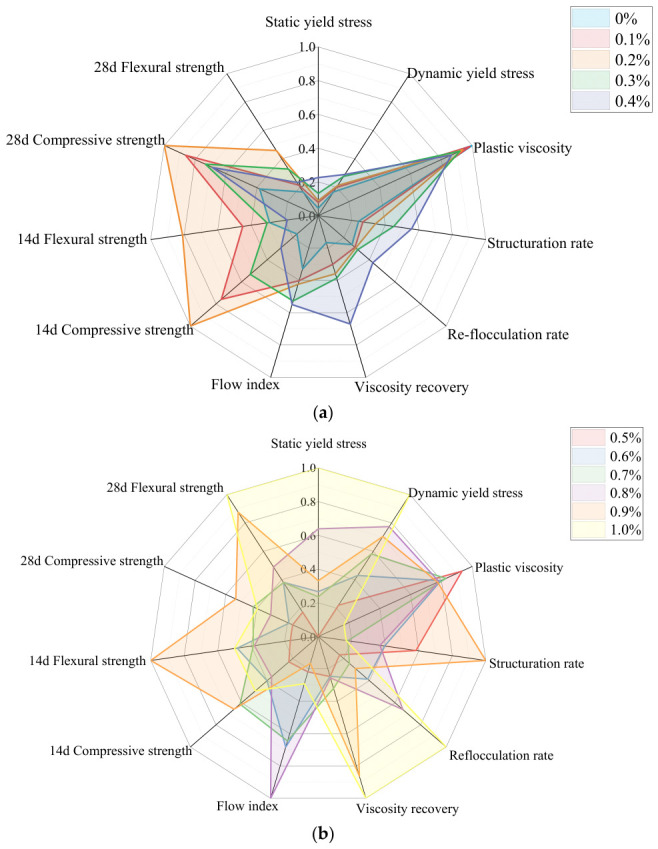
Normalised radar plots of steel fibre-reinforced mortar in different fibre dosage: (**a**) 0–0.4%; (**b**) 0.5–1.0%.

The radar plots in Figure 15 present the multi-dimensional performance of mixtures at different fibre contents, integrating rheological parameters (yield stress, viscosity-related indices, thixotropy) and mechanical properties (compressive and flexural strength in different curing ages). A clear shift in dominant performance characteristics can be observed with increasing fibre dosage. At low fibre contents (≤0.2%), the radar profiles are relatively balanced, with moderate values across most parameters. In this region, improvements are mainly observed in mechanical performance, particularly compressive and flexural strength, while rheological parameters remain within a workable range. This indicates that low fibre dosages primarily enhance structural performance without significantly compromising flow behaviour.

At intermediate fibre contents (approximately 0.3–0.5%), the radar shapes become less uniform, reflecting increased variability among parameters. In particular, reductions in flow index and structuration-related parameters are observed, indicating instability in flow behaviour and structural rebuilding. This region corresponds to the previously identified transition zone, where fibre dispersion and interaction begin to interfere with both rheology and mechanical performance.

At higher fibre contents (≥0.8%), the radar plots expand significantly along rheological axes, particularly static yield stress, dynamic yield stress, re-flocculation rate and thixotropy. This indicates that steel fibres strongly enhance structural build-up, rapid recovery, and resistance to deformation. Among all parameters, thixotropy and yield stress exhibit the most pronounced increase, confirming that steel fibres primarily act as a structural modifier in the fresh state. In contrast, plastic viscosity and flow index show more complex behaviour, suggesting that excessive fibre content may introduce flow resistance and instability during extrusion.

The radar analysis highlights that the most sensitive parameters to steel fibre incorporation are those associated with structural build-up and recovery, namely yield stress and thixotropy-related indices. These parameters govern the transition between fluid-like and solid-like behaviour and are directly linked to buildability and shape retention in 3D printing. Mechanical properties, particularly flexural strength, also show significant enhancement at higher fibre contents, reflecting improved crack-bridging capacity and fibre–matrix interaction. Overall, the radar plots demonstrate that steel fibres have a multi-scale influence on cementitious systems, with the most significant impact observed in rheological parameters controlling structural stability. This suggests that optimisation of fibre dosage should prioritise balancing yield stress and thixotropic behaviour with acceptable flow characteristics, while simultaneously leveraging the benefits in flexural performance.

### 4.2. Composite Index

In order to provide an integrated assessment of the coupled rheological and mechanical behaviour, a composite performance index was introduced based on the normalised parameters. The composite index was calculated as the arithmetic mean of all normalised parameters, assigning equal weighting to each indicator. This approach was adopted to provide a transparent and unbiased basis for comparing the relative performance of the mixtures, without introducing subjective assumptions regarding the importance of individual parameters [64].

**Figure 16:**
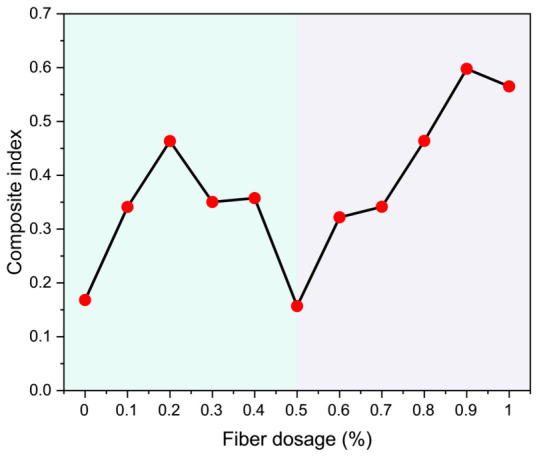
Composite index of different steel fibre-reinforced mortar.

The variation in the composite index with steel fibre dosage is presented in Figure 16. At lower fibre contents (≤0.5%), the composite index increases from 0.17 for the reference mixture to a maximum of 0.46 at 0.2%, followed by a slight reduction and stabilisation in the range of 0.35–0.36 at 0.3–0.4%. A significant drop to 0.16 is observed at 0.5%, indicating a temporary deterioration in overall performance, consistent with the previously identified instability in both rheological and mechanical properties at this dosage. The peak at 0.2% suggests that this fibre content provides the most balanced combination of improved mechanical strength and controlled rheological behaviour within the low-dosage regime. At higher fibre contents (>0.5%), the composite index increases again, reaching 0.46 at 0.8% and attaining a maximum value of 0.60 at 0.9%, before slightly decreasing to 0.57 at 1.0%. This increase is primarily driven by the significant enhancement in rheological parameters, particularly yield stress, re-flocculation rate, and thixotropy recovery, which dominate the composite index due to their large relative gains at high fibre dosages.

However, it should be emphasised that the composite index serves as a comparative evaluation tool rather than a direct measure of printability. Because all parameters were assigned equal weighting, substantial gains in rheological properties contribute positively to the index, even when such increases may become detrimental to practical printing operations. Excessively high rheological resistance can increase pumping pressure requirements, reduce extrudability and increase the risk of blockage during material delivery. Consequently, the highest composite index values obtained at 0.8–1.0% do not necessarily indicate the optimum mixtures for extrusion-based 3D concrete printing. Instead, the results suggest that these mixtures are dominated by rheological enhancement at the expense of processability. In contrast, the 0.2% steel fibre mixture achieved a comparable composite index while maintaining a more balanced combination of mechanical enhancement and rheological performance. This dosage provided sufficient structural build-up and recovery without introducing excessive flow resistance.

### 4.3. Working Window and Printability Implication

The composite index analysis in Section 4.2 shows that steel fibre dosage substantially changes the balance between rheological resistance and mechanical enhancement. However, printability cannot be defined by the composite index alone, because extrusion-based 3D concrete printing also requires stable pumping, continuous extrusion, and sufficient shape retention after deposition. Therefore, the composite index was further interpreted together with the individual rheological parameters to establish a practical working window for fibre dosage selection and material design.

The investigated system can be regarded as a high-strength printable mortar, as the reference mixture achieved a 28-day compressive strength of 82.6 MPa and the strongest mixture reached 116.0 MPa. Therefore, the proposed working window is most applicable to high-performance printable mortar systems incorporating Portland cement, limestone filler, fine aggregate smaller than 2 mm, water-reducing admixtures, viscosity-modifying admixture and short straight steel fibres. Although the exact base mix proportions are not disclosed due to industrial confidentiality, the performance-based ranges proposed here provide useful guidance for similar high-strength printable cementitious materials.

**Figure 17:**
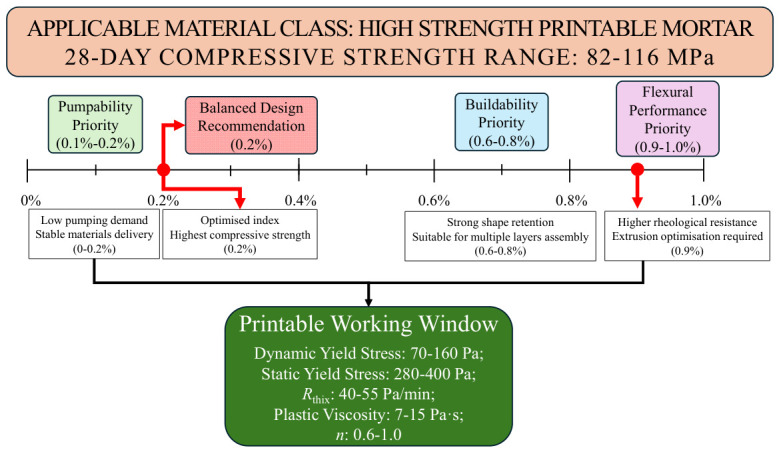
Printable working window of steel fibre-reinforced cementitious materials.

Based on the combined rheological and mechanical results, a practical printable working window is proposed in Figure 17. For the material investigated in this study, mixtures with static yield stress of approximately 280 to 400 Pa, dynamic yield stress of 70 to 160 Pa, plastic viscosity of 7 to 15 Pa·s, re-flocculation rate of 40 to 55 Pa/min, and flow index between 0.6 and 1.0 showed the most favourable balance between pumpability, extrudability, buildability and strength development. Within this range, the material can maintain adequate structural build up after deposition without developing excessive resistance during pumping and extrusion.

The working window also indicates that different steel fibre dosages may be suitable for different printing priorities. When pumpability and process stability are the main requirements, fibre dosages of 0.1 to 0.2% are more appropriate because they provide mechanical improvement while maintaining relatively low rheological resistance. For general large-scale 3D concrete printing, 0.2% steel fibre is recommended as the most balanced dosage. This mixture achieved the highest compressive strength at both 14 and 28 days, maintained improved flexural performance and remained within the proposed printable window. It also provided the highest composite index among mixtures with fibre contents not exceeding 0.5%, indicating a favourable balance between fresh and hardened performance.

When buildability and shape retention are prioritised, such as in taller elements or geometrically demanding components, fibre dosages of 0.6 to 0.8% may be more suitable. These mixtures exhibited higher yield stress and stronger structural recovery, which can improve resistance to deformation after deposition. However, they may require adjustment of printing speed, nozzle geometry, or pumping pressure to maintain continuous extrusion. For applications where flexural performance and post-cracking resistance are the dominant requirements, fibre dosages of 0.9 to 1.0% may be considered, as these mixtures provided the highest flexural strength. Nevertheless, these high fibre contents also generated strong rheological resistance and are therefore less suitable for routine large-scale printing unless the printing system is specifically optimised for high-resistance materials.

In addition, the behaviour at 0.5% fibre dosage should be interpreted as a local fluctuation rather than a definitive optimum or failure point. Several rheological and mechanical parameters showed reductions around this dosage, suggesting that the interaction between steel fibres and the cementitious matrix is not fully monotonic. This may be related to local variations in fibre distribution and fibre interactions, although direct fibre dispersion measurements were not conducted in this study. The proposed working window provides a practical interpretation of the experimental results by linking fibre dosage, rheological response, mechanical performance and printing requirements. Rather than selecting steel fibre content based only on strength or composite index, the framework supports dosage selection according to the intended application. This provides a clearer route for translating laboratory rheological and mechanical data into material design guidance for steel fibre-reinforced 3D concrete printing.

### 4.4. Limitations and Future Work

Although this study provides a systematic evaluation of the rheological and mechanical behaviour of steel fibre-reinforced cement-based materials, several limitations should be acknowledged. First, fibre dispersion, orientation and clustering were not directly quantified. The discussion of fibre distribution was inferred from the observed rheological fluctuations and mechanical responses, particularly the non-monotonic behaviour around 0.5% fibre dosage. Therefore, this interpretation should be regarded as a possible explanation rather than direct evidence. Future work should incorporate X-ray computed tomography, image-based analysis or sectioned specimen observation to quantify fibre spatial distribution, orientation and clustering. This would allow direct correlation between fibre arrangement, rheological response and load transfer efficiency.

Second, the mechanical specimens used in this study were mould-cast and therefore do not capture extrusion-induced fibre alignment, layer interfaces or anisotropic mechanical behaviour. In real extrusion-based 3D concrete printing, steel fibres may become preferentially oriented along the printing direction due to nozzle flow, which can improve strength in one direction while reducing performance in others. The selected pre-shear protocol was adopted to homogenise the mixtures, minimise differences in shear history and provide a controlled basis for comparing rheological parameters among different fibre dosages. However, this protocol cannot fully reproduce the complex shear field, fibre alignment and confinement effects generated during pumping and nozzle extrusion. Future studies should therefore compare mould-cast and printed specimens in different loading directions to evaluate anisotropy, fibre orientation and interlayer performance.

Third, the proposed printability implications were derived from rheological measurements and mechanical performance rather than direct printing trials. While the results provide useful guidance for pumpability, extrudability and buildability, full-scale printing introduces additional process variables, including pumping pressure, hose length, nozzle geometry, printing speed, layer height, deposition interval and environmental conditions. Future work should validate the proposed 0.2% dosage and the higher-dosage application ranges through controlled printing trials. Key parameters should include extrusion continuity, filament geometry, surface quality, build height, deformation after deposition, interlayer bonding strength and printing-induced fibre orientation.

Finally, the working-window framework proposed in this study should be further refined by coupling material rheology with process parameters. Future research should establish quantitative relationships between yield stress, plastic viscosity, thixotropic recovery, printing speed, nozzle size, pumping pressure and layer stability. In addition to laboratory-scale extrusion and multi-layer stacking tests, industrial-scale printing trials are recommended to evaluate material performance under practical production conditions, including long-distance pumping, continuous extrusion, nozzle movement, layer deposition and interlayer bonding. Such work would enable the development of predictive material-process compatibility models and provide more robust guidance for the industrial application of steel fibre-reinforced materials in large-scale 3D concrete printing.

## 5. Conclusions

This study systematically evaluated the coupled effects of steel fibre incorporation on the rheological behaviour, mechanical performance and potential printability of cement-based materials for extrusion-based 3D concrete printing. The measured rheological indicators provide a basis for interpreting the potential printability implications of the mixtures, including pumpability, extrudability, buildability and shape retention. The main conclusions are summarised as follows:

- Steel fibre addition significantly modified the rheological response of the mixtures. Static yield stress increased from 261.2 Pa for the reference mixture to 733.6 Pa at 1.0% fibre dosage, while dynamic yield stress increased from 59.3 Pa to 264.0 Pa. These results indicate that higher fibre contents enhance structural build up and flow resistance, which may improve shape retention but can also increase pumping and extrusion difficulty.
- Plastic viscosity increased from 4.6 Pa·s for the reference mixture to 36.8 Pa·s at 1.0% fibre dosage, indicating substantially higher internal friction at high fibre contents. The flow index varied from 0.52 to 1.43, showing that steel fibres changed not only the magnitude of rheological parameters but also the flow regime. Most mixtures remained shear-thinning, while the 0.8% mixture showed clear shear-thickening behaviour.
- Thixotropy-related parameters were also affected by fibre dosage. The re-flocculation rate increased from 40.4 Pa/min for the reference mixture to 93.9 Pa/min at 1.0%, while viscosity recovery increased from 2320.0 Pa·s to 8703.7 Pa·s. These results suggest that steel fibres can promote rapid structural recovery after shear, which is beneficial for potential filament stability after deposition. However, excessive recovery may also reduce extrusion continuity and interlayer bonding potential.
- Compressive strength exhibited a non-monotonic trend with increasing fibre dosage. The highest compressive strength was achieved at a fibre dosage of 0.2%, reaching 90.3 MPa at 14 days and 116.0 MPa at 28 days, compared with 64.1 MPa and 82.6 MPa for the reference mixture at 14 and 28 days, respectively. This corresponds to an increase of approximately 41% at 14 days and 40% at 28 days, demonstrating that a low steel fibre dosage can significantly enhance matrix performance without causing excessive disruption. Flexural strength benefited more strongly from higher fibre contents. The 28-day flexural strength increased from 10.8 MPa for the reference mixture to 15.4 MPa at 0.9% and 16.2 MPa at 1.0%. This confirms the effectiveness of steel fibres in improving crack bridging and post-cracking resistance, although these higher dosages were also associated with greater rheological resistance.
- The composite performance index increased from 0.17 for the reference mixture to 0.60 at 0.9% and 0.57 at 1.0%, mainly due to strong rheological enhancement and flexural improvement at high fibre contents. However, these high dosages may not be suitable for routine extrusion-based printing because the increased yield stress, viscosity and recovery response may reduce pumpability and extrusion stability.
- Considering both the composite index and the proposed printable working window, the 0.2% fibre mixture provided the most balanced performance for potential large-scale 3DCP application. This dosage achieved the highest compressive strength, improved flexural strength and maintained moderate rheological resistance, with static yield stress of 283.5 Pa, dynamic yield stress of 70.7 Pa, plastic viscosity of 6.9 Pa·s, re-flocculation rate of 42.6 Pa/min and a flow index of 0.65.
- The results indicate that steel fibre dosage must be selected according to application priority. Low dosages around 0.1 to 0.2% are more suitable where pumpability and process stability are required, 0.2% provides the best overall balance for potential large-scale printing, 0.6 to 0.8% may be useful where higher buildability is prioritised, and 0.9 to 1.0% may be considered for flexural performance-driven applications if the printing system is adapted to accommodate higher rheological resistance.

Future work should validate these findings through laboratory-scale extrusion tests, multi-layer stacking trials, interlayer bonding evaluation and industrial-scale printing trials under practical production conditions.

## Figures and Tables

**Table 2:** The chemical compositions of the cement and limestone filler.

Binder Materials	Oxide (wt.%)									
	SiO_2_	Al_2_O_3_	CaO	Fe_2_O_3_	MgO	SO_3_	K_2_O	Na_2_O	HCI Insoluble Content	Loss on Ignition
Cement	19.8	4.9	62.6	2.3	2.6	3.1	0.7	0.2		1.65
Limestone filler	4.3		95.0	0.1					4.5	

**Table 3:** Steel fibre dosage for each mixture (vol.%).

Mix No.	1	2	3	4	5	6	7	8	9	10	11
Fibre ratio	0	0.1	0.2	0.3	0.4	0.5	0.6	0.7	0.8	0.9	1.0

**Table 4:** The summary of rheological results.

No.	Fibre Dosage (%)	Rheological Parameters						
		Static Yield Stress (Pa)	Dynamic Yield Stress (Pa)	Plastic Viscosity (Pa·s)	Structuration Rate (Pa/min)	Re-Flocculation Rate (Pa/min)	Viscosity Recovery (Pa·s)	Flow Index (*n*)
1	0	261.2	59.3	4.6	11.3	40.4	2320.0	0.52
2	0.1	277.9	67.3	5.1	11.9	42.1	3350.7	0.62
3	0.2	283.5	70.7	6.9	13.6	42.6	3799.0	0.65
4	0.3	304.7	86.2	7.6	16.2	43.7	4003.8	0.79
5	0.4	350.9	89.8	9.8	18.7	52.2	6175.9	0.82
6	0.5	239.6	72.7	7.1	19.3	33.4	2943.1	0.37
7	0.6	371.0	124.3	12.5	14.9	49.3	2928.2	1.00
8	0.7	356.1	161.1	11.2	9.8	39.0	3496.1	0.95
9	0.8	555.1	208.6	13.0	14.3	69.3	3003.3	1.43
10	0.9	403.0	191.5	13.3	29.1	42.3	7660.3	0.30
11	1.0	733.6	264.0	36.8	9.6	93.9	8703.7	0.47

**Table 5:** Summary of mechanical results.

No.	Fiber Dosage (%)	Mechanical Parameters			
		14 d Compressive Strength (MPa)	14 d Flexural Strength (MPa)	28 d Compressive Strength (MPa)	28 d Flexural Strength (MPa)
1	0	64.1	10.2	82.6	10.8
2	0.1	82.7	10.8	108.5	11.1
3	0.2	90.3	12.2	116.0	12.7
4	0.3	75.6	10.2	101.6	11.8
5	0.4	68.1	9.8	99.1	11.2
6	0.5	66.1	9.7	71.0	10.8
7	0.6	71.5	10.9	72.3	12.2
8	0.7	78.1	10.6	84.8	12.2
9	0.8	70.4	10.5	78.7	12.9
10	0.9	79.6	13.0	90.9	15.4
11	1.0	74.4	11.0	83.4	16.2

## Data Availability

The original contributions presented in this study are included in the article. Further inquiries can be directed to the corresponding authors.
